# Loss of ecologically important genetic variation in late generation hybrids reveals links between adaptation and speciation

**DOI:** 10.1002/evl3.187

**Published:** 2020-07-13

**Authors:** Greg M. Walter, Thomas J. Richards, Melanie J Wilkinson, Mark W. Blows, J. David Aguirre, Daniel Ortiz‐Barrientos

**Affiliations:** ^1^ School of Biological Sciences University of Queensland Brisbane 4072 Australia; ^2^ Current address: School of Biological Sciences Monash University Melbourne 3800 Australia; ^3^ Department of Ecology and Genetics Uppsala University Uppsala SE‐752 36 Sweden; ^4^ School of Natural and Computational Sciences Massey University Auckland 0745 New Zealand

**Keywords:** Adaptation, dominance, genetic incompatibilities, heterosis, hybrid sterility, intrinsic reproductive isolation, line cross analysis, natural selection, recombination, speciation, trade‐offs

## Abstract

Adaptation to contrasting environments occurs when advantageous alleles accumulate in each population, but it remains largely unknown whether these same advantageous alleles create genetic incompatibilities that can cause intrinsic reproductive isolation leading to speciation. Identifying alleles that underlie both adaptation and reproductive isolation is further complicated by factors such as dominance and genetic interactions among loci, which can affect both processes differently and obscure potential links between adaptation and speciation. Here, we use a combination of field and glasshouse experiments to explore the connection between adaptation and speciation while accounting for dominance and genetic interactions. We created a hybrid population with equal contributions from four contrasting ecotypes of *Senecio lautus* (Asteraceae), which produced hybrid genomes both before (F1 hybrid generation) and after (F4 hybrid generation) recombination among the parental ecotypes. In the glasshouse, plants in the second generation (F2 hybrid generation) showed reduced fitness as a loss of fertility. However, fertility was recovered in subsequent generations, suggesting that genetic variation underlying the fitness reduction was lost in subsequent generations. To quantify the effects of losing genetic variation at the F2 generation on the fitness of later generation hybrids, we used a reciprocal transplant to test for fitness differences between parental ecotypes, and F1 and F4 hybrids in all four parental habitats. Compared to the parental ecotypes and F1 hybrids, variance in F4 hybrid fitness was lower, and lowest in habitats that showed stronger native‐ecotype advantage, suggesting that stronger natural selection for the native ecotype reduced fitness variation in the F4 hybrids. Fitness trade‐offs that were present in the parental ecotypes and F1 hybrids were absent in the F4 hybrid. Together, these results suggest that the genetic variation lost after the F2 generation was likely associated with both adaptation and intrinsic reproductive isolation among ecotypes from contrasting habitats.

**Impact statement**

Evolutionary biologists have long sought to identify genetic mechanisms underlying adaptation and speciation to better understand how species diversify. Adaptation to contrasting environments occurs when different alleles are favored in different environments, which can lead to the accumulation of barriers to gene exchange. As speciation advances, reproduction between diverging populations will fail in any environment, creating an intrinsic barrier to interbreeding. Whether such intrinsic barriers arise as a consequence of the alleles responsible for adaptation to different habitats remains poorly understood. We created a late‐generation hybrid population by mating among four contrasting ecotypes of the same species. Intrinsic barriers to reproduction arose at the second generation as a dramatic reduction in fitness as a loss of fertility, but fitness was recovered the following F3 generation, suggesting that genetic variation causing the F2 reduction in fitness was lost in the subsequent generations. We tested the consequences of the lost genetic variation at the F2 generation by comparing the fitness of hybrid populations, before and after recombination, with the original ecotypes in their natural habitats. The F4 hybrid showed a reduction in phenotypic variation, exhibited reduced genetic variation for fitness in the natural habitats, and showed weaker fitness trade‐offs among habitats than the original ecotypes. Our results suggest that genetic variation underlying adaptation to contrasting environments also created intrinsic barriers to reproduction, which may underlie the early stages of divergence leading to speciation.

Natural selection acts largely upon the additive effects of genes to promote adaptation (Hill et al. 2008), and species can form when barriers to gene flow arise between populations adapting to contrasting environments (Coyne and Orr 2004). Thus, alleles beneficial in an environment promote adaptation, but whether the same alleles that underlie adaptation also create reproductive isolation among taxa remains an open question (Lowry et al. 2008; Baack et al. 2015; Fishman and Sweigart, 2018). Ultimately, we are interested in understanding whether genetic variation important for adaptation to different environments also contributes to the evolution of intrinsic reproductive isolation (Coyne and Orr 2004; Funk et al. 2006; Presgraves 2010; Moyle et al. 2012). If the additive effects of alleles that underlie adaptation are also involved in negative genetic interactions that cause hybrid failure, then we can better explore the genetic and ecological links between adaptation and speciation by identifying genetic variation common to both processes.

Adaptation and speciation have been linked by comparing the fitness and phenotype of artificial hybrids synthesized by mating populations adapted to contrasting habitats (Coyne and Orr 2004). Hybrid fitness will be determined by how the genomes of the parental taxa interact independent of environment, and whether the effect of such genomic interactions changes depending on which of the natural habitats they are tested in (Rundle and Whitlock 2001; Egan and Funk 2009). However, combining divergent genomes can affect the genetic composition of individuals in later generations in unexpected ways. Dominant alleles can create heterosis in hybrids, which can obscure patterns of adaptation and speciation (Lowry et al. 2008). Recombination can break associations among alleles, but this can result in a loss of genetic variation when derived alleles are genetically incompatible in the genetic background of other populations, creating intrinsic reproductive isolation as Bateson–Dobzhansky–Muller genetic incompatibilities (Bateson, 1909; Dobzhansky, 1937; Muller, 1942). Thus, genetic variation underlying patterns of adaptive divergence can be lost in hybrid generations due to heterosis and genetic interactions affecting fitness (Fenster and Galloway 2000b).

One method used to identify genetic differences between populations adapted to contrasting environments is the line‐cross analysis (also known as the joint‐scaling test), which compares the mean performance of two populations, and their hybrids, reciprocally transplanted into both the parental environments (Demuth and Wade 2005; Egan and Funk 2009; Richards et al. 2016). If additive gene action was responsible for adaptive divergence between the populations, then F1, F2, and backcross hybrids will have a predictable fitness value, known as the mid‐parent value, intermediate to the two parental populations (Fig. 1A). Dominant alleles that cause heterosis will cause the F1 to have greater fitness than that predicted by the mid‐parent value. Genetic interactions, created by recombination among derived alleles from the divergent populations, could have positive (e.g., adaptive introgression) or negative (e.g., genetic incompatibilities) effects on fitness, causing the fitness of the F2 and subsequent generations to deviate from the mid‐parent value, and the fitness of the F1 generation (Fig. 1A; Fenster and Galloway, 2000b).

**Figure 1:**
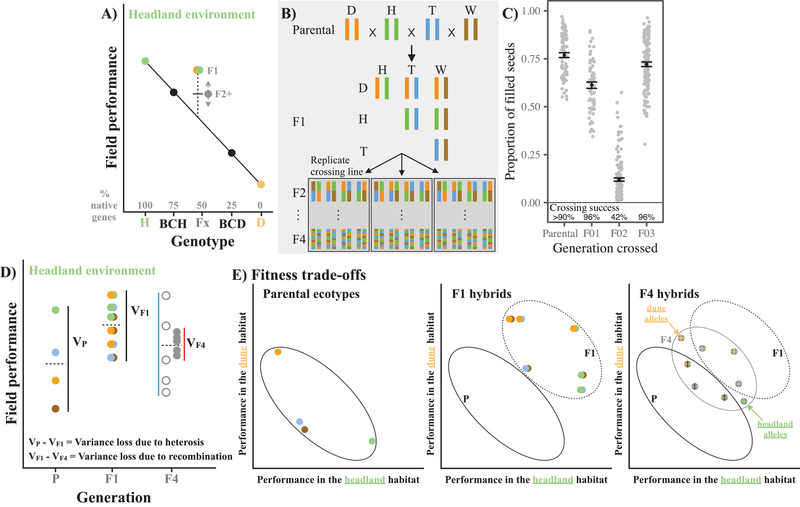
(**A)** Conceptual diagram predicting hybrid fitness for two populations. Panels (B) and (C) present our experimental design. Panels (D) and (E) extend the two‐population model (from **A**) for our experimental design with four populations. (**A)** Line‐cross analysis compares the mean fitness of two taxa in their native habitats. We present two ecotypes (colored circles) in one habitat, where pairs of colored circles represent their F1 crosses, black circles the backcrosses to each parental (BC) and gray circles the later generation hybrids. Under an additive model the hybrids will sit on the line connecting the two parents, which represents the midparent. Deviations of the F1 hybrid from the line are due to heterosis (dashed line). Deviations of later generation hybrids (F2+) from the intermediate between the midparent and the F1 are due to recombination and genetic interactions (gray arrows). (**B)** Our crossing design mated equally among all ecotypes to produce all combinations of F1 crosses, and then F2 hybrids with a grandparent from each ecotype. Replicate crossing lines were maintained independently after the F2 generation. (**C)** Loss of intrinsic fitness at the F2 generation. The number of successful crosses and the average number of fertile seeds per cross were substantially lower in the F2 generation. Gray circles represent all crosses, with the mean and one standard error represented by the solid circle with error bars. (**D and E)** We transplanted the parental ecotypes, F1 hybrids, and F4 hybrids into all four habitats and estimated variance in performance for each generation to test two hypotheses. (**D)** Differences in mean performance (dashed lines) reflect the same predictions as (A). Variance among the parental ecotypes (**V_p_**) represents all the genetic variance in field performance that could be inherited and maintained across generations under an additive model. However, similar to the two‐population model (**A**), high heterozygosity in F1 hybrids (**V_F1_**) should reduce variance due to the masking of recessive alleles. Hypothesis 1: Hybrid generations after recombination (**V_F4_**) could show an increase in variance (blue line and open circles), but if the reduction in fitness at the F2 generation removed ecologically important genetic variation, we would expected a decrease in variance (red line and filled circles). (**E**) Fitness trade‐offs are created when the parental ecotypes perform well in their native habitat, but poorly in foreign environments (here, for only the dune and headland habitats), creating a negative correlation between habitats (ellipse with solid line). F1 crosses with a native parent should have higher performance than F1 crosses with only foreign parents, maintaining the negative correlation (ellipse with broken line). For the F4 generation (gray ellipse), under an additive model, the negative correlation between habitats should be maintained by F4 genotypes that possess alleles from the native ecotypes (gray circles with colored lines representing a mix of the original ecotypes). Hypothesis 2: If the reduction in fitness at the F2 generation (from **D**) removed ecologically important genetic variation, we would expect fitness trade‐offs present in the parental ecotypes and F1 hybrids to be lost in the F4 hybrid, resulting in fitness correlations between habitats that are positive or zero for the F4 hybrid.

The line‐cross approach has been used effectively to identify whether adaptive divergence results in selection against hybrid genotypes, creating extrinsic postzygotic reproductive isolation (Rundle 2002; Egan and Funk 2009; Richards et al. 2016). However, within this framework, intrinsic versus extrinsic postzygotic reproductive isolation cannot be distinguished easily because reductions in fitness could be driven by the environment in which fitness is assayed. Building on the line‐cross approach, if it is possible to identify intrinsic reproductive isolation in the laboratory at the F1 or F2 generation, we can then compare the parents with later generation hybrids to identify how genetic variation lost following intrinsic reproductive isolation affects fitness for late generation hybrids tested in the natural habitats. If genetic variation linking adaptation and intrinsic reproductive isolation exists, then genetic variation removed due to its contribution to intrinsic reproductive isolation will have ecological consequences for later generation hybrids.

Here, we test whether we can detect genetic variation linking adaptation and speciation using four contrasting ecotypes within the adaptive radiation of an Australian wildflower species complex from the Asteraceae family (*Senecio lautus*). Ecotypes show strong phenotypic differences that reflect the environment they inhabit, which include coastal rocky headlands (Headland ecotype) and sand dunes (Dune ecotype), subtropical rainforest edges (Tableland ecotype), and dry sclerophyll woodland (Woodland ecotype) (Ali, 1969; Radford et al. 2004; Roda et al. 2013; Walter et al. 2016; Walter et al. 2018a). Ecotypes are self‐incompatible, possess the same number of chromosomes, and share generalist insect pollinators. Despite often being in close geographic proximity, there is no evidence of gene flow between populations of divergent ecotypes (James et al. 2020). Ecotypes are adapted to their contrasting habitats, and show strong fitness trade‐offs when transplanted into alternative habitats (Walter et al. 2016; Walter et al. 2018b).

We mated among the four ecotypes to create a late‐generation F4 hybrid (Fig. 1B), and tested the consequence of hybridization in laboratory and field experiments. To create the F4 generation, we combined all ecotypes equally (Fig. 1B). As predicted by models of outcrossing (Lynch, 1991), we observed strong heterosis in the performance of F1 hybrids when transplanted into the four natural habitats, but F1 crosses with a parent that was native to the transplant habitat performed better than F1 crosses with only foreign parents, suggesting that adaptive alleles were often dominant (Walter et al. 2016). In the laboratory, despite low hybrid lethality across all generations, we observed strong reductions in hybrid fertility at the F2 generation, followed by a complete recovery of fitness in the F3 generation (Fig. 1C; Walter et al. 2016). F2 Hybrid infertility indicated that recombination among genomes adapted to contrasting habitats creates negative genetic interactions among derived alleles that produces intrinsic reproductive isolation (Fenster et al. 1997; Li et al. 1997; Johansen‐Morris and Latta 2006). The fitness recovery in the F3 generation suggests incompatible ecotype‐specific alleles in the F2 generation were absent in the F3 generation (Erickson and Fenster 2006). We hypothesized that patterns of intrinsic F1–F3 hybrid fitness resulted from alleles that act additively within a population to promote adaptation, but also contribute to negative genetic interactions that create intrinsic reproductive isolation.

We used extensive field and glasshouse experiments to test the hypothesis that F2 hybrid failure was associated with a loss of genetic variation that was ecologically important in the natural habitats. To quantify changes in fitness in the natural habitats across the parental, F1 hybrid and F4 hybrid generations, we used a modified line‐cross experiment, where we shifted the focus to testing the changes in variance in fitness across generations. Under a null model, where dominance and genetic interactions do not affect variance in fitness across generations, we would expect that all genetic variation from the parental populations will be present in all hybrid generations (Lynch 1991; Fenster and Galloway 2000b), which would maintain constant variance in fitness across generations. However, given higher heterozygosity in the F1 hybrid generation, we expect that some fitness differences among the parents will be lost in the F1 generation (Fig. 1D, loss of variance from **V_P_** to **V_F1_**; Lynch and Walsh 1998). In addition, if negative genetic interactions that create intrinsic reproductive isolation involved alleles important for adaptation, then genetic variation underlying adaptation would not be present in the F4 generation and further fitness differences among parental ecotypes will be lost (Fig. 1D, loss of variance from **V_F1_** to **V_F4_**). We also expect that habitats with stronger natural selection for the native ecotype (i.e., show the strongest patterns of adaptation; Kawecki and Ebert 2004), would also show the greatest loss in fitness variation in the F4 hybrid because these habitats required more derived alleles across more loci that would be involved in negative genetic interactions underlying intrinsic reproductive isolation.

We also predicted that the loss of genetic variation after F2 hybrid failure would not only reduce variance in fitness, but also eliminate fitness trade‐offs in later hybrid generations. If alleles adapted to any particular environment were lost as a consequence of intrinsic reproductive isolation, then fitness trade‐offs that were present among habitats for the parental ecotypes would be retained in the F1 generation, but lost in the F4 generation (Fig. 1E). Therefore, by comparing changes in variance and changes in fitness trade‐offs across generations in a modified line‐cross design, it is possible to explore the connection between adaptation and speciation by testing whether the loss of genetic variation after intrinsic F2 hybrid failure has consequences for performance in the natural habitats.

## Methods

### CROSSING DESIGN

To create the F4 hybrid, we first sampled seeds from plants in one natural population from each of the four ecotypes, which we germinated and grew at the University of Queensland glasshouses. We sampled seeds for the Dune and Headland ecotypes at Lennox Head, NSW (−28.783005, 153.594018 and −28.813117, 153.605319, respectively), from the Tableland ecotype at O'Reilley's Rainforest Retreat, Qld (−28.230508, 153.135078), and the Woodland ecotype at Upper Brookfield, Qld (−27.479946, 152.824709). At each location, we collected seeds from 24 to 49 plants separated from each other by at least 10 m to minimize the likelihood of sampling close relatives. To grow plants, we scarified each seed and placed them in glass Petri dishes containing moist filter paper. After leaving them in the dark for 2 days, we transferred the germinated seeds to a 25°C constant temperature growth room with 12 h:12 h light:dark photoperiod. After 1 week, we transferred the seedlings to the glasshouse and transplanted them into 85 mm pots containing a mixture of 70% pine bark and 30% coco peat with slow release osmocote fertilizer and 830 g/m^3^ of Suscon Maxi insecticide. We conducted controlled crosses on mature plants by rubbing two mature flower heads together, labeling the flower heads, and collecting the seeds as they emerged.

We created the F4 ensuring each ecotype contributed equally and that at each generation (Fig. 1B), all full‐sibling families (hereafter, “families”) contributed equally to the next generation. First, we grew plants for the base population from seeds sampled from the natural populations and performed crosses among the ecotypes (*n =* 41–60 individuals/ecotype) to create all combinations of F1 hybrids (*n* = 12 crossing combinations; *n* = 20–25 families/cross type). We then mated among all combinations of crosses in the F1 generation such that all F2 families (*n* = 24 crossing combinations; *n* = 17–22 families/cross type) possessed a grandparent from each of the original parental ecotypes (e.g., F1_Dune,Headland_ × F1_Tableland,Woodland_). Given strong reductions in intrinsic fitness was observed in a previous Dune × Headland F2 hybrid (Walter et al. 2016), we maximized the number of F1 crosses to produce 458 F2 families in total. We then grew one individual from each F2 family. While F2 individuals showed no reduction in germination or growth when compared to the previous generation, we observed increased sterility (reduced crossing success as the percentage of crosses that produced more than one viable seed) and reduced fertility (49% reduction in seed set compared to F1 hybrids) when compared to previous generations (Fig. 1C; Walter et al. 2016). To replicate the construction of the F4 hybrid, we divided the viable F2 individuals into three replicate crossing lines. We then randomly mated among all F2 individuals within each line (*n =* 4–12 families/F2 cross type; total F2 families crossed *N* = 202) to produce the F3 generation (*N* = 259 families). To produce the F4 generation, we grew one individual from each family from the F3 generation, and randomly designating each individual as a sire or dam. We then mated 115 sires to 114 dams in a full‐sibling, half‐sibling crossing design to produce 198 F4 generation families. The numbers of families and individuals used to create each generation are listed in Table S1.

We conducted two experiments using the F4 hybrid. In experiment 1, we grew the F4 hybrid alongside the parental populations in the glasshouse to quantify differences in morphology. In experiment 2, we transplanted seeds of the parental, F1 and F4 generations into the four natural habitats to compare differences in fitness. In two previous papers, we presented analyses of the field performance data for the parental ecotypes and F1 hybrids (Walter et al. 2016; Walter et al. 2018b). In the current study, we build on the previous work by presenting analyses of new morphological data (experiment 1), and by re‐analyzing the published data on the parental ecotypes and F1 hybrids while including new data on the F4 hybrid field performance (experiment 2).

### EXPERIMENT 1: GLASSHOUSE PHENOTYPES

To measure morphological traits in the parental and F4 generations, we grew four individuals from each full sibling family of the F4 (*n* = 198 full‐sibling families, total *N* = 770 individuals) in 30 cell growth trays containing the same potting media described above. Alongside the F4, we grew four individuals from 23 to 27 full sibling families for each of the parental ecotypes (*N* = 366 individuals). Plants were grown in a 25°C controlled temperature room with a 12 h:12 h day:night photoperiod. After 8 weeks of growth, we measured plant height and sampled one fully mature leaf for each plant. We used the software “*Lamina*” to analyze the scanned leaf and quantify six variables relating to leaf size and leaf shape (Bylesjo et al. 2008). Using the outputs of Lamina, we quantified leaf morphology using leaf area, leaf complexity $\left(\frac{leaf\;area^{2}}{leaf\;perimeter^{2}}\right)$, leaf circularity, number of indents standardized by leaf perimeter, and leaf indent width and depth.

### COMPARING F4 AND ECOTYPE MORPHOLOGY

To compare differences in multivariate phenotype between the F4 and parental ecotypes, we used a multivariate analysis of variance (MANOVA) on the seven morphological traits measured in experiment 1. We first standardized all morphological traits to a mean of zero and standard deviation of one before including them as a multivariate response variable. To test whether the F4 was phenotypically different to each ecotype, we conducted a separate MANOVA for each pairwise comparison between the parental ecotypes, and the F4. We used a Bonferroni corrected α‐value of 0.0125 (α = 0.05/*n*, where *n* represents the number of tests). To visualize differences among all ecotypes and the F4, we estimate **D**, the variance–covariance matrix representing multivariate phenotypic divergence. To do so, we first conducted another MANOVA that included all parental ecotypes (but not the F4). From this, we extracted the sums of squares and cross‐product matrices for the ecotypes (SSCP_H_) and error terms (SSCP_E_) to calculate their mean‐square matrices by dividing by the appropriate degrees of freedom (MS_H_ = SSCP_H_/3; MS_E_ = SSCP_E_/365). Using the mean‐square matrices, we calculated **D** = (MS_H_ – MS_E_)/*nf*, where *nf* represents the number of measured individuals per genotype in an unbalanced design, calculated using equation 9 in Martin et al. (2008). Our D‐matrix then represents divergence in multivariate mean phenotype, among the parental ecotypes, after removing the residual phenotypic variation. To visualize the phenotypic space occupied by the F4 relative to the parental ecotypes, we decomposed **D** into orthogonal axes (eigenvectors) and calculated the scores for the first two eigenvectors for all ecotypes, and the F4.

### EXPERIMENT 2: FIELD TRANSPLANT

To compare field performance of the parental, F1, and F4 generations, we transplanted seeds of each generation into all four natural habitats. For the F4 generation, we planted 18 seeds from each full‐sibling family at each transplant site divided equally among six experimental blocks (habitat *n* = 3532–3582 seeds, total *N* = 14,265 seeds). Alongside the F4 hybrid, we transplanted seeds from 30 families of each of the four parental populations (*n* = 180 seeds/ecotype/habitat), and from 30 families for each of the six F1 crosses (*n* = 180 seeds/F1 cross/habitat). Therefore, we transplanted a total of 7670 seeds from the parental ecotypes and 4311 F1 hybrid seeds (Total *N* = 21,453 seeds). For a detailed description of the field experiment, see Walter et al. (2016; 2018b). Briefly, we glued each seed to a toothpick using non‐drip glue and planted them in 25 mm × 25 mm plastic grids in March 2014. To replicate natural germination conditions, we suspended shadecloth (50%) 15 cm above each experimental block and watered them daily for 3 weeks. During the initial 3‐week period we measured emergence and mortality daily. Following the initial 3 weeks, we measured survival at weeks 4, 5, 7, and 9, and then monthly until 20 months at which time fewer than 20% of germinated plants remained, and we ceased the experiment. We recorded fitness as: whether each seedling emerged, whether each seedling reached 10 leaves (as a measure of seedling establishment) and produced a bud (reached maturity). All measures of fitness were collected as binary data.

### IMPLEMENTATION OF BAYESIAN MODELS

In the subsequent analyses, we implemented Bayesian models to analyze field fitness in experiment 2. We implemented models to compare mean field performance of the F4 with the parental ecotypes and F1 hybrids, and then to quantify fitness trade‐offs among transplant habitats for the parental ecotypes. The Bayesian models are described below, and were implemented using R (R Core Team, 2019) within the package “*MCMCglmm*” (Hadfield, 2010). From each model, we extracted 2000 Markov chain Monte Carlo (MCMC) samples, which provided the posterior distribution for the parameters of interest. The chains of all models were run for four million iterations, with a burn‐in of 200,000 iterations and a sampling interval of 2000 iterations. We checked model convergence by ensuring autocorrelation was below 0.05, and making sure the effective sample size for each parameter exceeded 85% of the number of posterior samples specified. We used uninformative parameter expanded priors and checked their sensitivity by re‐implementing all models while adjusting the parameters and ensuring the posterior distribution did not change.

### QUANTIFYING CHANGES IN MEAN FITNESS ACROSS GENERATIONS

To estimate mean fitness for the parental ecotypes, and F1 and F4 hybrid generations transplanted into all four habitats, we used MCMCglmm to implement

(1) $$y_{ijklm}=H_{i}+C_{j}+H_{i}\times C_{j}+f_{l\left(j\right)}+b_{k\left(i\right)}+e_{m\left(ijkl\right)},$$

where transplant habitat ($H_{i}$), parental/F1/F4 cross type ($C_{j}$), and their interaction ($H_{i}\times C_{j}$) were included as fixed effects. Block within transplant habitat ($b_{k(i)}$) and full‐sibling family within each parental/F1/F4 cross type ($f_{l(j)}$) were included as random effects, and $e_{m(ijkln)}$ represents the model error. We implemented Equation 1 with seedling emergence, seedling establishment, and plant maturity as a multivariate response variable ($y_{ijklm}$) with a logit link function.

### QUANTIFYING CHANGES IN VARIANCE IN FITNESS ACROSS GENERATIONS

To identify how natural selection differed among habitats, and how this changed between the parental, F1, and F4 generations, for each generation we estimated the variance in fitness, and the covariance in fitness among the four habitats. We used a character state approach, where different environments represent different traits. We used the field performance of the parental ecotypes, and the F1 and F4 hybrids and implemented

(2) $$y_{ijklm}=H_{i}+c_{j}+f_{k\left(j\right)}+b_{l\left(i\right)}+e_{m\left(ijkl\right)},$$

where transplant habitat ($H_{i}$) was included as a fixed effect. To estimate a (co)variance matrix for field fitness at each generation, we applied Equation 2 to the parental, F1, and F4 generations separately. We included family ($f_{k(j)}$) and block within habitat ($b_{l(i)}$) as random effects, with $e_{m(ijkl)}\;$ representing the residual error. Fitness variance in each habitat was quantified as the among‐genotype variance in field performance, but the genotypes of interest were different each generation because each generation was represented by a different genetic make‐up. The parentals were represented by the four populations of parental ecotypes, the F1 hybrids by the six types of F1 crosses, and the F4 by the full‐sibling families. To estimate variance among the four ecotypes for the parents, and to estimate the variance among the six F1 crosses, we included $c_{j}$ as a random effect for these generations, which represented the different populations for the parental ecotypes, and the different cross‐types for the F1 hybrid generation. For the F4 generation, we were only concerned with among‐family variance.

For each random effect in Equation 2, we estimated a 4 × 4 covariance matrix representing the fitness variance in each habitat, and the fitness covariance among habitats. We implemented Equation 2 with a residual covariance matrix that estimated different variances in each habitat, but with residual covariances fixed at zero because individuals (seeds) could not be planted in two habitats simultaneously. Because fitness trade‐offs in the parental ecotypes were strongest at seedling establishment (Walter et al. 2018b), we only applied Equation 2 to seedling establishment, which we included as a binary univariate response variable ($y_{ijklm}$).

### QUANTIFYING CHANGES IN FITNESS TRADE‐OFFS ACROSS GENERATIONS

To quantify fitness trade‐offs among transplant habitats, we converted the covariance matrices (estimated using Equation 2) into correlation matrices representing the correlation in performance between habitats, for each generation. A negative fitness correlation between two habitats represents a fitness trade‐off because genotypes (e.g., the parental ecotypes) perform well in their native habitats, but poorly in other habitats.

To quantify changes in fitness trade‐offs across generations, we compared the parental, F1, and F4 correlation matrices using a covariance tensor approach (Hine et al. 2009; Aguirre et al. 2014). Unlike most methods that only allow pairwise comparisons of matrices, the benefit of the tensor approach is that it allowed us to examine differences among all three correlation matrices simultaneously. We only provide a brief description (and supplementary code) of the tensor approach here, but refer readers to more detailed descriptions of the mathematics and the application to quantitative genetics studies in Basser and Pajevic (2007), Hine et al. (2009), Aguirre et al. (2014), and Walter et al. (2018a). The tensor approach is a geometric approach founded on the diagonalization of symmetric matrices (i.e., eigenanalysis), and is used to calculate a set of (scaled) orthogonal axes known as eigentensors that describe coordinated changes in the elements of the original matrices being compared. Briefly, a symmetrical matrix is constructed, the S‐matrix, whose elements describe element‐by‐element variation among the original matrices. Diagonalization of **S** finds the axes (eigenvectors) along which the elements of the original matrices differ the most. The eigenvectors of **S** are scaled and arranged to construct the eigentensors, which are used to understand how each of the original matrices and variables (in our case, the four habitats) contribute to the differences among matrices captured by the eigentensors.

The matrix coordinates for eigentensors are used to identify how each of the original matrices contributes to the differences among matrices described by the eigentensors. The coordinates are linear combination scores calculated for each matrix and each eigentensor, and can be interpreted in the same way as principal component scores—larger values indicate a stronger correlation of the matrix with the eigentensor, and in this context, a greater contribution of a particular matrix to the differences among matrices captured by the eigentensor. We predicted that if fitness trade‐offs were lost in the F4 hybrid, then the coordinates for the first eigentensor of the parental ecotype and F1 hybrid matrices would be similar to each other, but different to the F4 hybrid. Such differences in the coordinates would indicate that the parental ecotypes and F1 hybrid showed very different patterns of fitness among habitats compared to the F4 hybrid.

The second step in the approach is to uncover the variables (i.e., the variables defining the original matrices, which in our case were the four different habitats) that contribute most strongly to the differences among matrix elements captured by an eigentensor. We achieve this through analysis of the eigentensors, which can be interpreted in exactly the same way as eigenvectors in conventional approaches such as principal components analysis: variables with large absolute loadings are strongly associated with the eigenvector, and the sign of the loadings (positive or negative) indicate whether variables are responding in the same or opposing directions. In our particular context, loadings of an eigenvector (of an eigentensor) with the same sign indicate a consistent directional change in the strength of fitness trade‐offs across generations (e.g., correlations between habitats became less negative across generations). By contrast, loadings with different signs indicate that as some correlations between habitats changed in one direction (e.g., became more negative) other genetic correlations changed in the opposite direction (e.g., became more positive). We can then use matrix projection to determine how strongly the original matrices were associated with particular eigenvectors of eigentensors that describe changes in magnitude and orientation of trade‐offs across generations. We predicted that if negative fitness correlations among habitats (representing fitness trade‐offs) were lost in the F4 generation, then eigenvectors (from the first eigentensor) that describe a change in the strength of fitness trade‐offs (i.e., have strong loadings with the same sign) would show similar values for the parental and F1 matrices, but different values for the F4 matrix.

## Results

### LEAF MORPHOLOGY OF THE F4 HYBRID WAS INTERMEDIATE TO THE PARENTAL ECOTYPES

**Figure 2:**
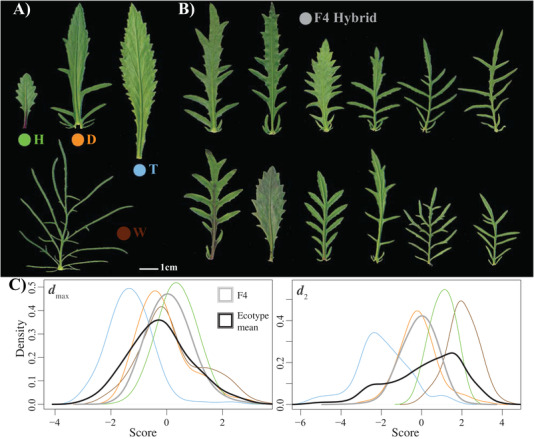
Morphological variation in the ecotypes, and the F4 hybrid. (**A)** Ecotypes vary dramatically in leaf morphology. (**B)** The F4 exhibited large variation in leaf morphology, visually intermediate among the original ecotypes. (**C)** The distribution of ecotype and F4 scores for the first two axes of (**D)**, which shows the F4 (gray lines) occupying an area in phenotypic space similar to the mean of all ecotypes (black lines) but lacking the phenotype extreme present in the parental ecotypes (colored lines).

Ecotypes show strong differences in leaf morphology (Fig. 2A), with the F4 hybrid exhibiting phenotypes intermediate to the parental ecotypes (Fig. 2B). Pairwise MANOVAs showed that the leaf morphology of the F4 hybrid was significantly different to all parental ecotypes (Dune: Wilks’ λ = 0.71, *F*_1,857_ = 50.782, *P *< 0.001; Headland: Wilks’ λ = 0.64, *F*_1,863_ = 69.747, *P *< 0.001; Tableland: Wilks’ λ = 0.38, *F*_1,866_ = 192.36, *P *< 0.001; Woodland: Wilks’ λ = 0.22, *F*_1,864_ = 445.1, *P *< 0.001). The MANOVA conducted on the parental ecotypes described a significant difference in multivariate mean phenotype (Wilks’ λ = 0.03, *F*_3,362_ = 117.86, *P *< 0.001). We used the MANOVA to calculate **D**, the covariance matrix representing among ecotype divergence in mean phenotype. The first two axes of **D** (***d***_max_ and ***d***_2_) described 84% and 14% of phenotypic divergence, respectively. ***d***_max_ captured differences between the Tableland and Headland ecotypes, with ***d***_2_ capturing differences between the Woodland and Tableland ecotypes (Fig. 2C). The mean phenotype of the F4 hybrid was intermediate and most similar to the Dune ecotype, but lacked the extreme phenotypes of the Headland, Tableland, and Woodland ecotypes.

### MEAN FITNESS IN THE NATURAL HABITATS REVEALED PATTERNS OF ADAPTATION AND PERSISTENT HETEROSIS

**Figure 3:**
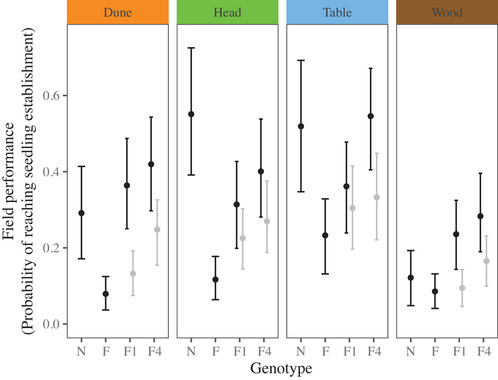
Mean performance of foreign (F) and native (N) parental ecotypes, and the F1 and F4 hybrids in each habitat. Credible intervals represent the 95% Highest Posterior Density (HPD) intervals. Presented in gray for the F1 is the mid‐parent of all ecotypes, and for the F4, the intermediate between the mid‐parent and the F1. Genetic interactions had positive fitness effects on the F4 hybrid in all habitats.

As reported previously (Walter et al. 2018b, 2016), we found strong patterns of adaptation where native ecotypes performed better than foreign ecotypes in all four transplant environments (Fig. 3). Results for maturity were similar (Fig. S1). As evidence of heterosis, F1 hybrids performed better than the mid‐parent of all ecotypes, especially in the dune and woodland habitats. Importantly, F1 crosses with a native mother (i.e., native cytoplasm) performed similarly to the reciprocal cross, suggesting that cytoplasm did not determine F1 fitness (Fig. S2). Under an additive model, where genetic interactions have no effect on mean fitness, we expect the F4 hybrid to perform intermediate to the value between the F1 hybrid and parental mid‐parent. We found that in all transplant habitats, the F4 hybrid performed better than expected (Fig. 3), suggesting that heterosis persisted after several generations of recombination and that genetic interactions after the F2 generation have an overall positive effect on fitness in this system.

### VARIANCE IN FITNESS IN THE NATURAL HABITATS WAS LOST IN THE F4 HYBRID

**Figure 4:**
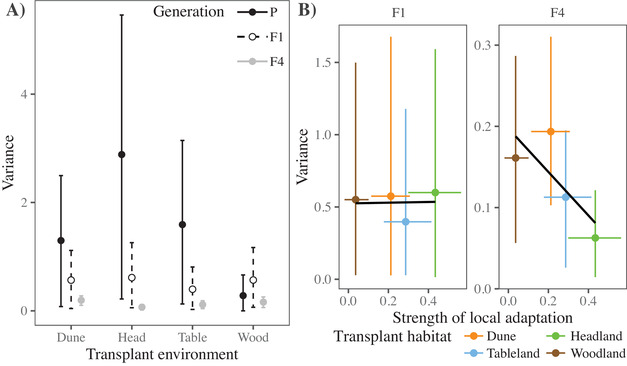
Variance in field performance for all generations transplanted into the four natural habitats. Credible intervals represent 95% HPD intervals. (**A**) Compared to the parental ecotypes and F1 hybrids, variance in field performance was lowest in the F4 hybrid. Filled circles and solid lines represent the parentals, unfilled circles and broken lines the F1 hybrid, and gray circles and lines, the F4 hybrid. (**B**) Stronger patterns of adaptation were negatively associated with the amount of variance in the F4 hybrid, but not the F1 hybrids. Strength of adaptation was quantified as the difference in fitness between the native and foreign ecotypes. Estimating separate regression slopes for each MCMC iteration showed that the distribution of the slope was negative and did not overlap zero at 86% HPD for the F4 hybrid.

Under an additive model of gene action, we would expect all genetic variation present in the parental ecotypes to also be present in the F4 generation (Fig. 1D). However, given we observed a reduction in intrinsic fitness at the F2 generation and strong ecotype‐specific heterosis for F1 field performance, we may expect that some of this variation may have been lost. Differences between the parental and F1 generation represents changes due to dominant alleles in one ecotype masking recessive alleles of other ecotypes, while differences between the F1 and F4 generations will be due to recombination and negative genetic interactions removing genetic variation. We found that, except for the woodland habitat, variance in field fitness reduced from the parental ecotypes to the F4 hybrid, with the F1 exhibiting intermediate variance (Table 1 and Fig. 4A). Therefore, variance among the parental ecotypes in each habitat was successively lost in each hybrid generation.

**Table 1:** Variance‐covariance matrices for field performance of: **(A)** the parental ecotypes, **(B)** F1 hybrids, and **(C)** F4 hybrids. The diagonals, denoted in gray, represent variance in fitness in each habitat. Covariances between habitats are located above the diagonal, with correlations presented below the diagonal. Numbers in parentheses denote the 95% HPD credible intervals. Later generation hybrids have lower variance in fitness, and have lost the strong negative genetic correlations in fitness between habitats present in the parental and F1 generations

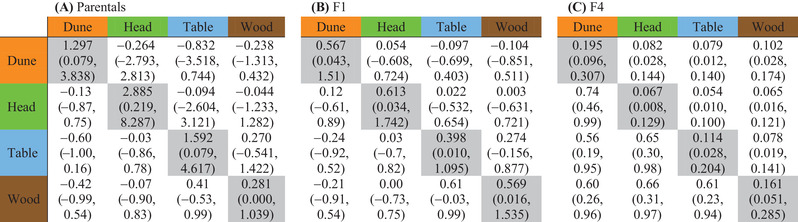	

#### Stronger patterns of adaptation reduce variance in fitness

If the genetic variation lost after the reduction in F2 hybrid fertility was adaptive in each of the original ecotypes, we predicted that the transplant environments that showed strong native‐ecotype advantage for the parental ecotypes (Fig. 3) would also show reduced variance in fitness for the F4 hybrid. Consistent with our prediction, the habitats associated with stronger native‐ecotype advantage also showed much lower variance in F4 hybrid fitness (Fig. 4B). Specifically, we found a significant negative correlation between the strength of adaptive divergence in each habitat (Fig. 3; nativeecotype performance–foreignecotype performance) and the amount of variance in the F4 generation (Fig. 4B). By contrast, the F1 generation did not show this trend, suggesting that recombination after the F1 generation was responsible for the loss of variation.

### FITNESS TRADE‐OFFS WERE LOST IN THE F4 HYBRID

If the genetic variation removed after the F2 fitness reduction not only reduced fitness variance in the natural habitats (previous section), but also removed fitness trade‐offs among the natural habitats, we predicted that we would observe fitness trade‐offs in the parental ecotypes and F1 ecotypes, but these trade‐offs would be weakened or lost in the F4 hybrid. Negative correlations between transplant habitats provide evidence of fitness trade‐offs because genotypes change relative fitness between habitats. For example, native ecotypes perform relatively well in their native habitat, but relatively poorly in foreign habitats, creating a negative fitness correlation between native and foreign habitats. We found evidence of strong negative correlations among habitats for the parental ecotypes, and weaker negative correlations among habitats for the F1 hybrid (Table 1). By contrast, the F4 hybrid exhibited strong positive correlations among all habitats (Table 1), suggesting that the F4 hybrids lost the fitness trade‐offs present in both the parental ecotypes and F1 hybrids.

**Figure 5:**
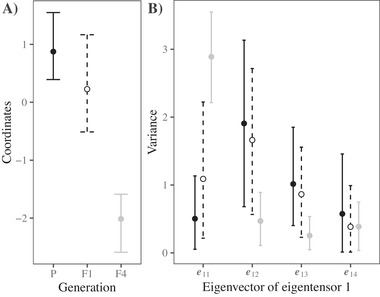
The covariance tensor captured changes in among‐habitat fitness correlations across generations. The first eigentensor describes 66.8% of the total differences among the generations (see Table 2). All credible intervals represent 95% HPD intervals. (**A**) The coordinates of each matrix in the space of the first eigentensor suggests that the differences captured by the first eigentensor are due to differences between the F4 hybrid, and the parental ecotypes and F1 hybrid. (**B**) Projecting the eigenvectors describing the first eigentensor, through the original matrices, quantified how each generation contributed to the differences described by the first eigentensor. The first eigenvector, representing differences in the magnitude of fitness trade‐offs across generations, separates the F4 hybrid from the other generations, suggesting that the F4 hybrid lost the fitness trade‐offs present in the parental ecotypes and F1 hybrid. The next two eigenvectors represent changes in fitness correlations between habitats, which also describe large differences between the F4 hybrid and the other generations. (see Table 2 for the loadings of each vector).

To quantify the changes in fitness trade‐offs across generations, we used a covariance tensor, which compared fitness correlations among habitats, across all three generations (Table 1). Differences in the coordinates represent differences among the original matrices that underlie the differences described by an eigentensor. As predicted, the coordinates separated the F4 hybrid from the parental ecotypes and F1 hybrid, suggesting that fitness correlations between habitats were different in the F4 hybrid (Fig. 5A). Therefore, the fitness trade‐offs present in the parental ecotypes and F1 hybrid were significantly reduced in the F4 hybrid (Table 1). The first eigenvector (of the first eigentensor) captures the greatest differences among the original matrices, and is described by habitat loadings that are all strong and in the same direction (Table 2), suggesting that this eigenvector describes changes in the magnitude of fitness trade‐offs across generations. Projecting the first eigenvector (of the first eigentensor) through the original matrices showed that, as predicted, the eigenvector representing a change in the magnitude of fitness trade‐offs also described differences between F4 hybrid, and the parental ecotypes and F1 hybrid (Fig. 5B). Therefore, fitness trade‐offs present in the parental ecotypes and F1 hybrid were absent in the F4 hybrid.

**Table 2:** Tensor analysis comparing the parental, F1 and F4 correlation matrices in Table 1. We present only eigentensor 1, which described the greatest difference among the original matrices (α = 2.608, HPD interval: 0.813, 4.438). λ represents the eigenvalue associated with each eigenvector (columns), with the proportion representing the proportion of the differencedescribed by each eigenvector. Loadings in bold represent strong contributions of the habitat to describing the eigenvector

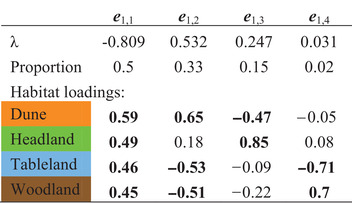	

## Discussion

Here, we mated equally among four contrasting ecotypes and tested the consequences of F2 hybrid sterility for phenotype and fitness in the F4 hybrid. We predicted that if genetic variation lost after the F2 fitness reduction was ecologically important, then we would observe reductions in variance in field fitness and a loss of trade‐offs present in the parental ecotypes and F1 hybrids. In the glasshouse, the F4 hybrid that was phenotypically intermediate, but lacked the phenotypic extremes that make each ecotype phenotypically unique (Fig. 2). In the natural habitats, the F4 hybrid displayed heterosis despite several generations of recombination (Fig. 3). We observed a substantial reduction in variance in fitness from the parental ecotypes to the F4 generation (Fig. 4A), which was strongest in the habitats that showed stronger patterns of adaptation (Fig. 4B). Furthermore, fitness trade‐offs, quantified by negative genetic correlations in fitness among habitats, were present in the parental and F1 generations, but were absent in the F4 hybrid (Fig. 5). Together, our results suggest that dominant alleles created negative genetic interactions at the F2 generation that removed phenotypic variation in the glasshouse, and removed genetic variation underlying adaptation and fitness trade‐offs among contrasting habitats.

### IDENTIFYING GENETIC VARIATION CONNECTING ADAPTATION AND SPECIATION

Our results connect adaptation and speciation by providing empirical evidence that genetic variation associated with adaptation was also associated with intrinsic reproductive isolation. Adaptation can be viewed as a build‐up of beneficial alleles maintained by natural selection whose additive effects enhance fitness in the local environment (Johansen‐Morris and Latta 2006). Intrinsic reproductive isolation arises when derived alleles, unique to different populations, are combined and create negative genetic interactions that reduce hybrid fitness independent of the environment (Moyle et al. 2012; Sweigart and Flagel, 2015; Fishman and Sweigart, 2018). Therefore, our data suggest that reductions in F2 hybrid fertility removed alleles that act additively within populations to promote adaptation, but that negatively interact between ecotypes, favoring the transition from ecotypes to species.

The F4 hybrid was phenotypically intermediate but more similar to the Dune ecotype (Fig. 2), which is the closest to the suspected ancestor‐like form of these ecotypes. Phenotypic variation and variation in fitness in the natural habitats was lost from the more derived forms (Headland and Tableland ecotypes). Therefore, similar to a recent study (Matute et al. 2020) that showed a removal of derived alleles after extensive hybridization, we believe that negative genetic interactions remove derived alleles and caused the hybrid to revert to a state similar to the common ancestor. However, our results also reveal the importance of understanding dominance for combining divergent genomes. Similar to other studies (e.g., Fenster and Galloway 2000a), we found that combining populations from contrasting habitats created persistent heterosis in later generation hybrids. The traditional explanation is that heterosis is created by dominance combined with the escape from inbreeding and genetic drift (Lynch 1991).

Persistent heterosis could also, in part, explain the problem of “general vigor,” where late‐generation hybrids perform well in all habitats and display positive fitness correlations among habitats, despite fitness trade‐offs in the parental taxa (Fry 1993). The observation of both fitness trade‐offs and heterosis during the early stages of speciation may be a consequence of the combined contributions of: (1) genetic drift and inbreeding creating lower fitness genotypes in the population, and (2) alleles underlying adaptation that create fitness trade‐offs among habitats. As favorable alleles rise in frequency during adaptation, their beneficial effects on fitness would be countered by the deleterious effects of inbreeding and random genetic drift, which would make it difficult for populations to reach an adaptive peak. This hypothesis requires testing, but could provide support for the role of drift in allowing populations to shift between adaptive peaks during the early stages of adaptive radiation (Wright 1982).

Our approach cannot identify the allelic variants that pleiotropically contribute to both adaptation and intrinsic reproductive isolation. However, our study justifies future genetic experiments that can identify whether alleles underlying fitness in the natural habitats pleiotropically contribute to both adaptation and intrinsic reproductive isolation, or whether different alleles underlie each process and are connected by physical linkage or linkage disequilibrium. For instance, it was previously thought that alleles underlying adaptation of *Mimulus guttatus* to toxic levels of copper also created genetic incompatibilities (Macnair and Christie 1983), but further experiments revealed that it was tight linkage between different alleles underlying each process (Wright et al. 2013). Molecular mapping techniques (e.g., Quantitative Trait Loci mapping) should be combined with large‐scale field transplants of F2 hybrids to identify the loci underlying fitness in each habitat and quantify whether these loci also underlie the intrinsic fitness reduction in the laboratory.

### DOMINANT ALLELES PROMOTE ADAPTATION BUT ALSO CONTRIBUTE TO INTRINSIC REPRODUCTIVE ISOLATION

**Figure 6:**
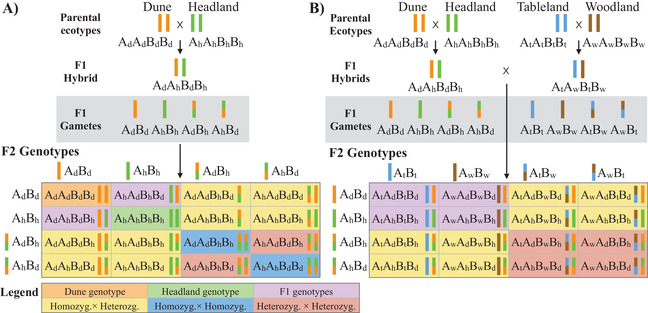
Two loci models identifying the genetic basis of intrinsic reduction in F2 fitness. Colored bars represent chromosomes from a specific ecotype, and subscript lettering represents the ecotype the allele is from. Models assume alleles at both loci are derived and unique to each ecotype. (**A)** The representation of a traditional F2 hybrid created by mating two taxa. Demuth and Wade (2005) showed that the most common F2 genotypes involve a heterozygote, creating negative homozygote × heterozygote (yellow cells), or heterozygote × heterozygote (red cells) genetic interactions between derived alleles. Parental genotypes that have no negative fitness effects (orange and green cells) are as frequent as negative homozygous × homozygous interactions (blue cells), cancelling out their negative fitness effects. (**B)** Extending the model to four ecotypes is complicated, and we can only present a simple example. Because we equally combined the four ecotypes, we always mated unrelated F1 hybrids to produce the F2 hybrid. Reductions in fitness can be explained by negative homozygote × heterozygote and heterozygote × heterozygote genetic interactions as they are the most common F2 genotypes.

Our experiments also indicate an important genetic architecture of speciation and adaptation: dominant alleles that contribute to adaptation within species may also lead to negative genetic interactions between ecotypes resulting in intrinsic reproductive isolation (Li et al. 1997; Demuth and Wade 2005). When we created the F2 hybrids for the current study, we mated unrelated F1 hybrids (e.g., F1_Dune,Headland_ × F1_Tableland,Woodland_), which increased the heterozygosity of our F2 hybrids compared to traditional F2 hybrids created by mating two parental taxa (Fig. 6A). F2 sterility was severe, suggesting that the initial recombination between chromosomes from different ecotypes created negative interactions among dominant alleles that were largely responsible for genetic incompatibilities and that reduced intrinsic fitness in the F2 hybrid (Fig. 6B). Importantly, the same dominant alleles could not reduce F1 fitness because it was not the presence of the dominant alleles that reduced fitness, but the effect of recombining derived dominant alleles from divergent populations, which created negative genetic interactions among loci that reduced F2 hybrid fitness (Fenster et al. 1997; Demuth and Wade 2005). Dominant alleles, compared to recessive alleles, escape demographic sinks when they are rare and are more likely to be favored by natural selection (Haldane 1924). Thus, our results suggest that the fixation of dominant alleles promoted adaptation and either had pleiotropic effects that caused intrinsic reproductive isolation, or were linked to alleles that did so (Li et al. 1997; Demuth and Wade 2005).

Ultimately, we cannot dismiss the effect of negative interactions between the cytoplasm and nucleus as a cause for reductions in F2 hybrid fitness (Burton et al. 2013), which has been shown in other systems including within Asteraceae (Sambatti et al. 2008). However, because cytoplasm did not contribute to F1 fitness in the natural habitats in the current study (Fig. S2), and because native versus foreign cytoplasm in backcross genotypes had little effect on fitness in the natural habitats (Melo et al. 2014; Richards et al. 2016), we favor the conclusion that negative nuclear genetic interactions were largely the cause of reductions in F2 hybrid fitness (Edmands 1999).

### USING QUANTITATIVE GENETICS TO LINK ADAPTATION AND SPECIATION

Although relatively underutilized in studies of intrinsic reproductive isolation, the study of quantitative genetic variation can help us understand how genetic incompatibilities arise among populations. For instance, recent work on the genetics of intrinsic reproductive isolation suggests that derived alleles responsible for genetic incompatibilities are not always fixed in different populations and are segregating as polymorphisms in natural populations (Cutter 2012; Sweigart and Flagel 2015; Larson et al. 2018). To test whether derived alleles that underlie adaptation also contribute to reproductive isolation before they are fixed, future studies could create hybrid populations and track additive genetic variance for both fitness and phenotype across generations. Such an approach surveys the polymorphic effects of adaptation on reproductive isolation across the genome. This kind of information can reveal the tempo and mode of speciation, perhaps hinting rapid evolution of reproductive isolation if genetic variation that promotes adaptation also contributes to hybrid sterility even during the early stages of adaptation when the alleles are still segregating in a population and are not yet fixed.

Phenotypically, the F4 hybrid was intermediate between all ecotypes, but lacked the phenotypic extremes of the Headland, Tableland, and Woodland ecotypes. The loss of phenotypic extremes has two potential explanations. First, if the phenotypes of the original ecotypes require particular combinations of alleles, then the polygenic nature of quantitative traits will make it difficult for recombination to assemble the particular combinations required to produce the phenotype of the original ecotypes. However, if the dominant alleles lost due to intrinsic reproductive isolation also contributed to the phenotypes of the original ecotypes, then it is possible that the loss of ecotype‐specific dominant alleles removed the phenotypic variation present in the original ecotypes. It is likely that both explanations contributed to the loss of phenotypic extremes in the F4 hybrid. In future, measuring phenotypes of all generations could identify the dominant phenotypes by comparing parental ecotypes to the F1 hybrids, and whether it is the dominant phenotypes that are lost in the F4 generation after intrinsic reproductive isolation.

## Conclusion

Our work indicates that the genetic variation involved in negative genetic interactions (that created genetic incompatibilities) among ecotypes was likely involved in adaptation to their respective habitats. We can then view the evolution of ecotypes from a perspective where selection acts upon additive genetic variation by increasing allele frequencies at independent loci (Hill et al. 2008) that can be maintained by the evolution of limited recombination among loci in small populations, extensive maladaptive gene flow, or very strong selection for integrated phenotypes (Mayr 1954; Carson and Templeton 1984; Ortiz‐Barrientos et al. 2016). Some of the environment‐specific alleles that segregate within an ecotype fail in alternative genetic backgrounds of other ecotypes, creating intrinsic reproductive isolation leading to speciation.

## AUTHOR CONTRIBUTIONS

GMW, DOB, MWB, and JDA conceived and designed the study. GMW, TJR, and MJW collected the data, GMW analysed the data with input from JDA and MWB; all authors contributed to writing the manuscript.

## DATA ARCHIVING

All relevant data for our analyses can be accessed at Dryad: https://doi.org/10.5061/dryad.sbcc2fr3t.

## CONFLICT OF INTEREST

The authors declare no conflict of interest.

Associate Editor: S. Wright

## Supporting information


**Table S1**: Individuals and families used to create the F4.
**Fig S1**: Mean fitness in the natural habitats as the probability of reaching maturity.
**Fig S2**: Performance of the different cytoplasm for each side of the F1 crosses.

Supplementary Material
