# Supplementary material for: Loss of ecologically important genetic variation in late generation hybrids reveals links between adaptation and speciation
Source: Evol Lett. 2020 Jul 13;4(4):302–16. doi: 10.1002/evl3.187 (PMC7403682; doi:10.1002/evl3.187)
Supplement: Supplementary file 1 — Table S1: Individuals and families used to create the F4. Fig S1: Mean fitness in the natural habitats as the probability of reaching maturity. Fig S2: Performance of the different cytoplasm for each side of the F1 crosses. [file EVL3-4-302-s001.docx]

**Supplementary material**

**Loss of ecologically important genetic variation in late generation hybrids reveals links between adaptation and speciation**

*Greg M. Walter^1,2 *^, Thomas J. Richards^3^, Melanie J Wilkinson^1^, Mark W. Blows^1^, J. David Aguirre^4^ and Daniel Ortiz-Barrientos^1^*

^1^University of Queensland, School of Biological Sciences, Brisbane 4072, Australia

^2^Current address: Monash University, School of Biological Sciences, Melbourne 3800, Australia

^3^Uppsala University, Department of Ecology and Genetics, Uppsala SE-752 36, Sweden

^4^Massey University, School of Natural and Computational Sciences, Auckland 0745, New Zealand

* Corresponding Author: Greg M. Walter
Email: [greg.walter@monash.edu](mailto:greg.walter@monash.edu)
Phone: +614 15 246 846

**Contents**

**Table S1:** Individuals and families used to create the ARF

**Fig S1:** Mean fitness in the natural habitats as the probability of reaching maturity

**Fig S2:** Performance of the different cytoplasm for each side of the F1 crosses

**Table S1:** Numbers of genotypes used to create the F4.

**Fig S1**: Mean performance in each habitat measured as the probability of reaching maturity. Patterns were similar for seedling establishment presented in the main text. Credible intervals represent the 95% Highest Posterior Density (HPD) intervals. **A)** Comparison of the F1 and F4 hybrids with foreign (F) and native (N) parental ecotypes. Presented in gray for the F1 is the mid-parent of all ecotypes, and for the F4, the intermediate between the mid-parent and the F1. Genetic interactions created positive fitness benefits for the F4 hybrid in all environments. **B)** The F1 hybrids with a native parent in each transplant habitat. Presented in gray is the mid-parent for each F1 cross type.

**
Fig S2:** Probability of reaching seedling establishment in each transplant habitat for both sides of each F1 cross. Credible intervals represent 95% HPD intervals for the estimate of mean field fitness. F1 crosses with native cytoplasm did not increase fitness, suggesting that cytoplasm did not determine F1 fitness in the natural habitats.
